# Epidemiology of Ebola Virus Disease in the Western Area Region of Sierra Leone, 2014–2015

**DOI:** 10.3389/fpubh.2017.00033

**Published:** 2017-03-02

**Authors:** Margaret Lamunu, Olushayo Oluseun Olu, James Bangura, Zabulon Yoti, Thomas Takpau Samba, David Kabba Kargbo, Foday Mohamed Dafae, Muhammad Ali Raja, Noah Sempira, Michael Lyazi Ivan, Aarti Sing, Fredson Kurti-George, Negusu Worku, Pamela Mitula, Louisa Ganda, Robert Samupindi, Roland Conteh, Kande-Bure Kamara, Beatrice Muraguri, Michael Kposowa, Joseph Charles, Malimbo Mugaga, Christopher Dye, Anshu Banerjee, Pierre Formenty, Brima Kargbo, Raymond Bruce Aylward

**Affiliations:** ^1^World Health Organization (WHO) Country Office, Freetown, Sierra Leone; ^2^World Health Organization (WHO) Country Office, Kigali, Rwanda; ^3^Ministry of Health and Sanitation, Freetown, Sierra Leone; ^4^World Health Organization (WHO) Headquarters, Geneva, Switzerland

**Keywords:** Ebola virus disease, outbreak, epidemiology, risk factors, urban setting, Western Area districts, Sierra Leone

## Abstract

**Introduction:**

Western Area (WA) of Sierra Leone including the capital, Freetown, experienced an unprecedented outbreak of Ebola from 2014 to 2015. At the onset of the epidemic, there was little information about the epidemiology, transmission dynamics, and risk factors in urban settings as previous outbreaks were limited to rural/semi-rural settings. This study, therefore, aimed to describe the epidemiology of the outbreak and the factors which had most impact on the transmission of the epidemic and whether there were different drivers from those previously described in rural settings.

**Methods:**

We conducted a descriptive epidemiology study in WA, Sierra Leone using secondary data from the National Ebola outbreak database. We also reviewed the Ebola situation reports, response strategy documents, and other useful documents.

**Results:**

A total of 4,955 Ebola cases were identified between June 2014 and November 2015, although there were reports of cases occurring in WA toward end of May. All wards were affected, and Waterloo Area I (Ward 330), the capital city of Western Area Rural District, recorded the highest numbers of cases (580) and deaths (236). Majority of cases (63.4%) and deaths (66.8%) were in WA Urban District (WAU); 44 cases were imported from other provinces. Only 20% of cases had a history of contact with an Ebola case, and more than 30% were death alerts. Equal numbers of males and females were infected, and very few cases (3.2%) were health workers. Overall, transmission was through contact with infected individuals, and intense transmission occurred at the community level. In WAU, transmission was mostly between neighbors and among inhabitants of shared accommodations. The drivers of transmission included high population movement to and from WA, overcrowding, fear and lack of trust in the response, and negative community behaviors. Transmission was mostly through contact and with limited transmission through sex and breast milk.

**Conclusion:**

The unprecedented outbreak in WA was attributed to delayed detection, inadequate preparedness and response, intense population movements, overcrowding, and unresponsive communities. Anticipation, strengthening preparedness for early detection, and swift and effective response remains critical in mitigating a potential urban explosion of similar future outbreaks.

## Introduction

Sierra Leone experienced a major and widespread outbreak of Ebola virus disease (EVD) between 2014 and 2015 (1). The Eastern Province of Sierra Leone was the first to be affected and, by August 2014, the disease had spread to the other regions of the country: Western Area (WA), Northern, and Southern Provinces. The outbreak resulted in an officially reported total number of 8,991 probable and confirmed cases and 3,955 deaths countrywide (2). The WA Region, comprising only 2 of the country’s 14 districts registered more than half of the reported cases and deaths.

To date, five species of the Ebola virus have been identified since the virus was discovered in 1976. It is the *Zaire ebolavirus (*ZEBOV), *Bundibugyo ebolavirus*, and *Sudan ebolavirus* that have so far caused dramatic outbreaks, mostly in Central and East Africa, and in South Africa, with high case fatality that ranged between 50 and 100% (3, 4). Gatherer (5) summarized eight separate outbreaks of ZEBOV reported between 1976 and 2008, each of which involved somewhere between 12 and 319 cases. As reported by Baize et al. (6), the outbreak in Sierra Leone, which occurred as an extension of the widespread 2013–2016 West African outbreak of Ebola, was due to ZEBOV.

The 2013–2016, West African outbreak of Ebola originated in Guinea and crossed geographical boundaries, affecting at least six African countries and more than three urban settings for the first time in history. Unlike previous Ebola outbreaks, which were mostly limited to rural areas or semi-urban settings, this epidemic introduced EVD into urban areas of WA, including Freetown Municipality (5). With close to 5,000 cases of Ebola, the outbreak in WA is almost 16 times more acute than any of the previous outbreaks attributed to ZEBOV, affecting about 12 times more people than the 2000–2002 Ebola outbreak in Uganda, which had previously been considered the worst Ebola outbreak on record (7).

At the onset of the epidemic, there was little information about the epidemiology, transmission dynamics, and risk factors in urban settings. It was believed that the urban and peri-urban context may itself be a factor that contributed to the unprecedented scale of the outbreak; indeed, the two major cities, Freetown Municipality and Waterloo Area 1 became major transmission hubs linked to cases elsewhere in the country and beyond. The objective of this study was, therefore, to describe the epidemiology of the disease in an urban setting of Sierra Leone including the factors which had the most impact on the transmission of the epidemic. Specifically, the study reviewed the drivers and patterns of transmission which may have resulted in the observed sustained transmission, and whether the drivers were different from those previously described in rural settings. The ultimate aim is to inform effective responses to future outbreaks of Ebola and other highly infectious diseases in similar urban settings, so as to be able to limit the excessive morbidity and mortality observed in this outbreak.

## Materials and Methods

### Study Design

We conducted a descriptive epidemiological study of the EVD outbreak in WA from 2014 to 2015. Quantitative and qualitative data for the study were obtained from the National EVD case-based database, an essential element of the active surveillance undertaken as part of the outbreak response; the district outbreak investigations and situation update reports; the district-specific response strategies; and other pertinent documents related to occurrence and reporting of cases and trends in WA.

### Description of the Outbreak Setting, WA Districts

Covering an area of 557 km^2^, WA is the smallest of the four administrative regions of Sierra Leone, yet the most densely populated, with an estimated population of about 1.4 million people (8). Western Area Urban (WAU) and Western Area Rural (WAR)—the two districts that constitute WA—comprise 12 cities/towns and 15 villages. The WA is further subdivided into 69 administrative wards: 20 wards in WAR (Wards 326–45) and 49 in WAU/Freetown Municipality (Wards 346–94). With an estimated population of 1.2 million people, WAU/Freetown Municipality is the largest city of Sierra Leone (9). It is home to virtually all ethnicities from all over the country with high population movement to and from the Provinces. In 2014, the population density of WAU was 1,224 people per square kilometer. Most people reside in informal settlements, in substandard living conditions, and with an average of 6–10 individuals in a single room. The region, like the rest of the country, is home to a young population, with a significant proportion (42%) below 15 years of age (9). Waterloo Area I is the capital for WAR, with good road connection to WAU and to elsewhere in the country.

### Study Method

### Case Definitions

Case identification was aided by the use of three EVD case definitions; suspect, probable, and confirmed case. A suspect case was defined as “any severely ill patient with history of fever 38.5°C of less than 3 weeks duration with vomiting and diarrhea, with or without any one of the following manifestations: abdominal pains, epistaxis, general body weakness, hematemesis, muscle or joint pain, hemoptysis, haemorrhagic or purpuric rash, blood in stool, haemorrhagic symptoms from any other site, conjunctival infections, and with no known predisposing factors for the haemorrhagic manifestations.” A probable case was defined as “any person meeting the suspected case definition criteria (above) and has had contact with a suspect, probable or a confirmed case/death within the previous 21 days of onset of disease symptoms; or any unexplained death.” A confirmed case was considered to be “any suspected or probable case that is laboratory confirmed by one or more of the following tests; detection of Ebola antigen in any body fluid, tissue, or clinical specimen by antigen detection enzyme-linked immunosorbent assay (ELISA) or immunohistochemistry; or demonstration of serum IgM or IgG antibodies by ELISA; or detection of Ebola nucleic acid by reverse-transcriptase polymerase reaction.”

Ebola alert cases were identified using EVD alert case definition which was “Fever and at least three of the following symptoms: vomiting, headache, nausea, diarrhea, difficulty breathing, fatigue, abdominal pain, loss of appetite, muscle or joint pain, unexplained bleeding, difficult swallowing, or hiccups.”

### Active Surveillance for Ebola in WA

**Figure 1:**
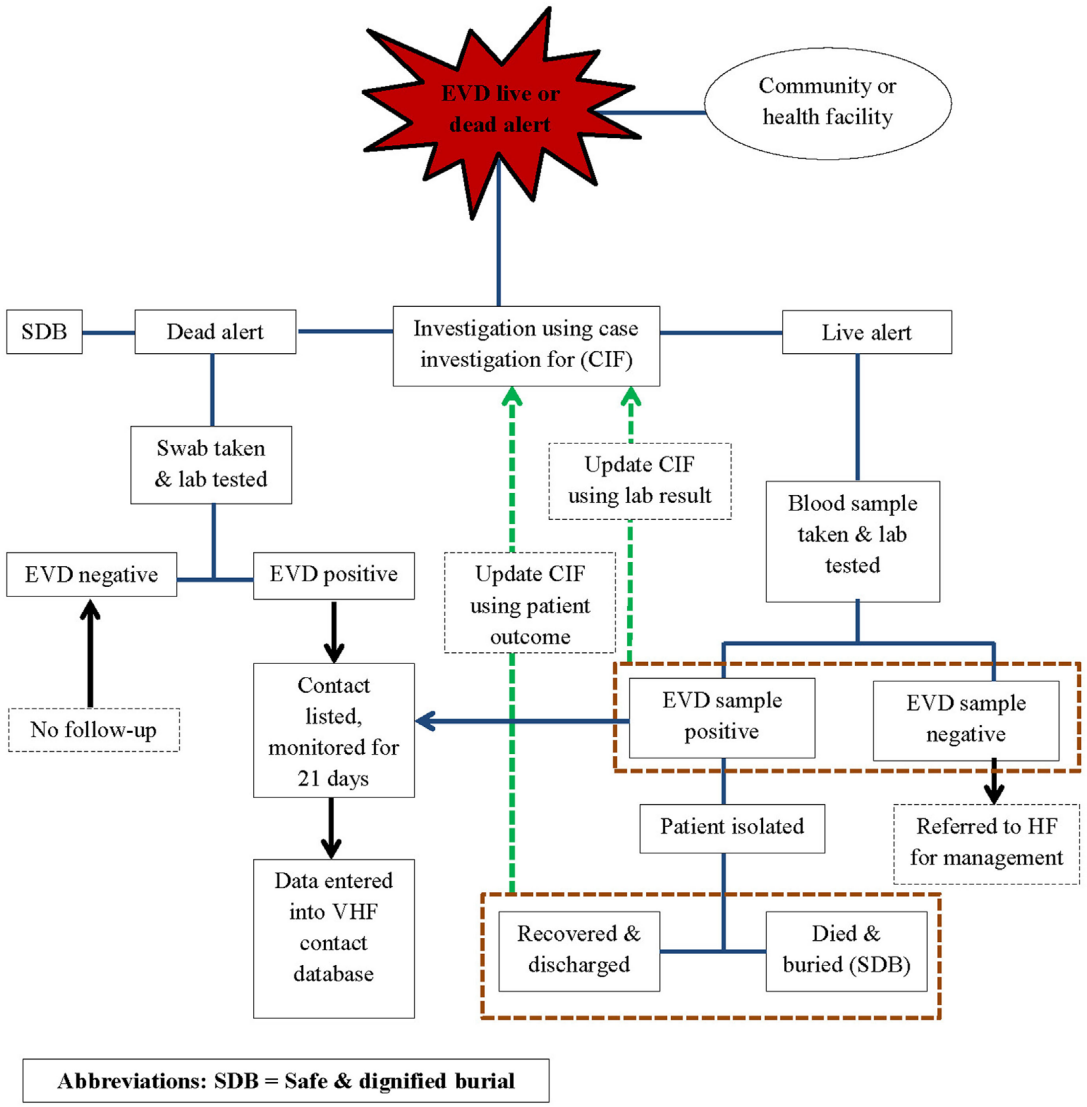
**Algorithm for Ebola outbreak surveillance in Western Area of Sierra Leone: 2014–2015**.

In line with the World Health Organization (WHO) recommendations for Ebola control, public health responders led by the national government and with technical support from WHO, the Centers for Disease Control and Prevention (CDC), the United Nations Population Fund (UNFPA), Médecins Sans Frontières (MSF), and other partners implemented an active surveillance system for EVD in WA during the outbreak response period. The active surveillance system was linked to the other components of the response system and entailed active case finding and alerting; case investigation, including laboratory testing of the alert cases for EVD; identification and listing of contacts of confirmed and probable EVD cases; and monitoring of high-risk contacts for a duration of 21 days. Alerts were derived from the EVD live and dead alerts (Figure 1).

Using the case definitions, trained community volunteers worked closely with district surveillance officers (DSOs) and epidemiologist to conduct active case finding and investigations in households, health facilities, and clinics. Any case fulfilling the alert case definition was referred to an Ebola treatment Centre (ETC), where samples were collected for testing. Swabs were routinely collected from all dead alert cases prior to a safe and dignified burial. Data were structured in the form of individual case information, based on information collected on site by way of a standardized case investigation form. The monitoring of contacts was done by way of a structured contact monitoring form. For each probable and confirmed case, collected data included geographical location, demographic information, case status on identification (dead or alive), exposure history, signs and symptoms, date of onset and date of reporting, laboratory results, admission status, and illness outcomes, including final case classification. EVD laboratories and the ETCs managed their respective data, but they shared critical information such as laboratory test results and patient outcomes with the surveillance team. This information was then used to update the individual case investigation forms, make final case classification, and update disease outcomes of probable and confirmed cases in the national EVD database. The implementation of the surveillance system was slow and under resourced in the beginning and was only improved and became fully functional from around March 2015.

At the height of the epidemic, when the caseloads overwhelmed existing capacities for response, all alert cases were considered suspect cases until they were tested. Suspect cases were discarded if negative, or reclassified as a confirmed EVD case following a positive test result. Individuals who were recorded as alert or suspect cases but died and were buried before sample collection for laboratory testing were classified as probable cases if they had a history of being in contact with a confirmed case of EVD. All contacts of probable and confirmed cases were recorded and monitored for a period of 21 days, the maximum duration of EVD incubation. To facilitate monitoring, movements of affected contacts were restricted through home quarantine of all households with confirmed or probable cases for 21 days. If households were overcrowded and associated with a potential risk of in-house transmission, offsite quarantine for high-risk contacts was implemented for the same 21-day period. Offsite quarantine meant that high-risk contacts were voluntarily and temporarily moved to a designated site, away from their household members, to facilitate monitoring and to prevent contamination of household members in the event of developing disease symptoms.

### Data Collection

For the quantitative component of the study, we extracted all EVD data for WA (for the period 2014–2015) from the national EVD database. We also reviewed selected national and WHO daily situation updates and investigation reports, including district-specific response strategy documents. In all the documents, we abstracted information for the characterization of the disease epidemiology, including drivers of transmission and propagation of the outbreak in WA. Key information sought included daily new cases, exposure history and contact information, how and when the case was identified, positive swabs, reports of secret burials, and missing contacts. We linked the information and used them to identify and deduce possible drivers of transmission during different phases of the outbreak. In case of information gap and possible clues, the abstracted information was verified and additional information sought from the DSOs.

### Data Analysis

Data analysis was carried out using Microsoft Excel and the Statistical Package for the Social Sciences (SPSS version 18). The first phase of the analysis involved determining what proportion of all alert cases identified in WA between June 1, 2014 and November 7, 2015 were classified as probable or confirmed EVD cases. Descriptive analysis of the EVD probable and confirmed cases was performed in terms of distribution of cases over time, and by location, demographic characteristics, history of exposure, and disease symptoms. Qualitative analysis entailed abstraction and synthesis of relevant information from available reports and documents.

### Ethical Considerations

The quantitative data for the study were collected as part of the active surveillance and epidemiological investigations of EVD alert cases, a component of the outbreak response interventions. Qualitative information was also generated during the outbreak response efforts. Clearance for the study and for its publication was obtained from the Ministry of Health and Sanitation (MOHS) in Sierra Leone, and WHO.

## Results

### General

A total of 37,847 alert cases were investigated during the epidemic period; 4,955 (13.1%) of them were classified as cases (3,966 confirmed and 989 probable). A total of 3,142 cases (63.4%) had been infected and detected in WAU/Freetown Municipality, 1,653 cases (33.4%) were from WAR, and 44 cases (0.9%) originated from the other 12 districts of Sierra Leone and were detected in WA (see Table 1). These data did not capture individuals potentially exposed and infected from WA and escaped to other Provinces before detection.

**Table 1:** **Classification status of Ebola virus disease (EVD) alert cases investigated by districts, Western Area, 2014–2015**.

	EVD status final case classification								
District of case	Total alerts	Probable cases (P)		Confirmed cases (C)		Not cases		Numbers/proportion of alerts by district that are EVD cases	
					
	*N*	*n*	%	*n*	%	*n*	%	*N* (*P* + *C*)	%
Western Area rural (WAR)	13,341	310	2.3	1,343	10.1	11,688	87.6	1,653	12.4
Western Area urban/Freetown Municipality (WAU)	23,640	647	2.7	2,495	10.6	20,498	86.7	3,142	13.3
Originating in one of 12 other districts	369	10	2.7	34	9.2	325	88.1	44	11.9
Unspecified location	496	22	4.4	94	19.0	380	76.6	116	23.4
Grand total	37,847	989	2.6	3,966	10.5	32,891	86.9	4,955	13.1

### Epidemic Detection and Recognition in WA

**Figure 2:**
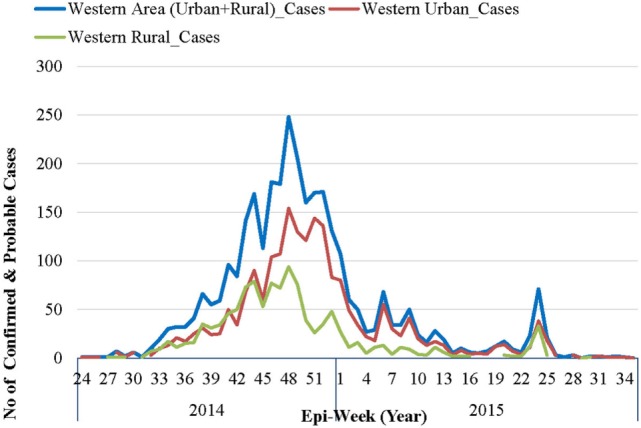
**Epidemic curve of confirmed and probable Ebola virus disease cases in Western Area: 2014–2015**.

The first official notification of the outbreak extension into WAU and Freetown Municipality was on July 12, 2014, following confirmation that a foreign national, an Egyptian who had traveled from Kenema, the largest city in the Eastern Province had checked himself into a private clinic (10). The epidemic curve, however, shows that the first EVD case in WAU/Freetown Municipality had a reported onset date in the second week of June 2014, while the first reported EVD case in WAR was recorded during the first week of July 2014 (see Figure 2). On the other hand, the National Ebola Response Centre (NERC) situation update report on 25th March 2015 indicated fairly high numbers of cases being recognized in WA as early as the end of May 2014. Retrospective verification and validation revealed that earlier cases occurred in Waterloo Area I (Ward 330) around May 2014, following an influx of high-risk contacts who had fled from Port Loko district and took refuge with relatives. Waterloo Area I is one of the 20 wards, the largest city, and is the district capital for WAR. This information was confirmed by one of the surveillance officers in WA, who reported that initial clusters of EVD in WA was in an area known as Fudia Terrace, in Money Bush in Waterloo Area I, WAR District. The disease had been introduced into this area through exposed contacts and symptomatic individuals fleeing from clusters of transmission in Port Loko District. The epidemic reportedly spread to WAU through movement of symptomatic individuals and high-risk contacts who went into hiding. While the above point to the fact that the epidemic in WA most likely started from Waterloo Area I in WA sometime in May 2014, it was difficult to identify the actual index case and when the case was introduced.

### Distribution of EVD Cases in the WA over Time

As per the EVD epidemic curve (see Figure 2), the outbreak lasted 14 months (56 epidemic weeks) in WAU and nine (9) months (36 epidemic weeks) in WAR. From the combined epidemic curve, the number of cases for WA increased exponentially from epi-week 34 and peaked in week 48 (November–December 2014). By the beginning of 2015, the weekly number of cases had dropped significantly, and there was sustained low-level transmission, resulting in a prolonged tail end of the epidemic curve. Smaller spikes were observed between epi-weeks 5 and 11 of 2015, and between epi-weeks 23 and 25 of 2015.

The epidemic curve in WAR followed a pattern similar to that of WAU, except for the size and duration of the epidemic. WAU experienced a sustained transmission and propagation of the epidemic, with varying intensities of transmission throughout the outbreak phase. WAR, on the other hand, experienced one major epidemic followed by two separate re-introductions of cases that had originated in WAU, in May and in July 2015.

### Geographic Distribution of EVD Cases and Deaths in WA, 2014–2015

**Figure 3:**
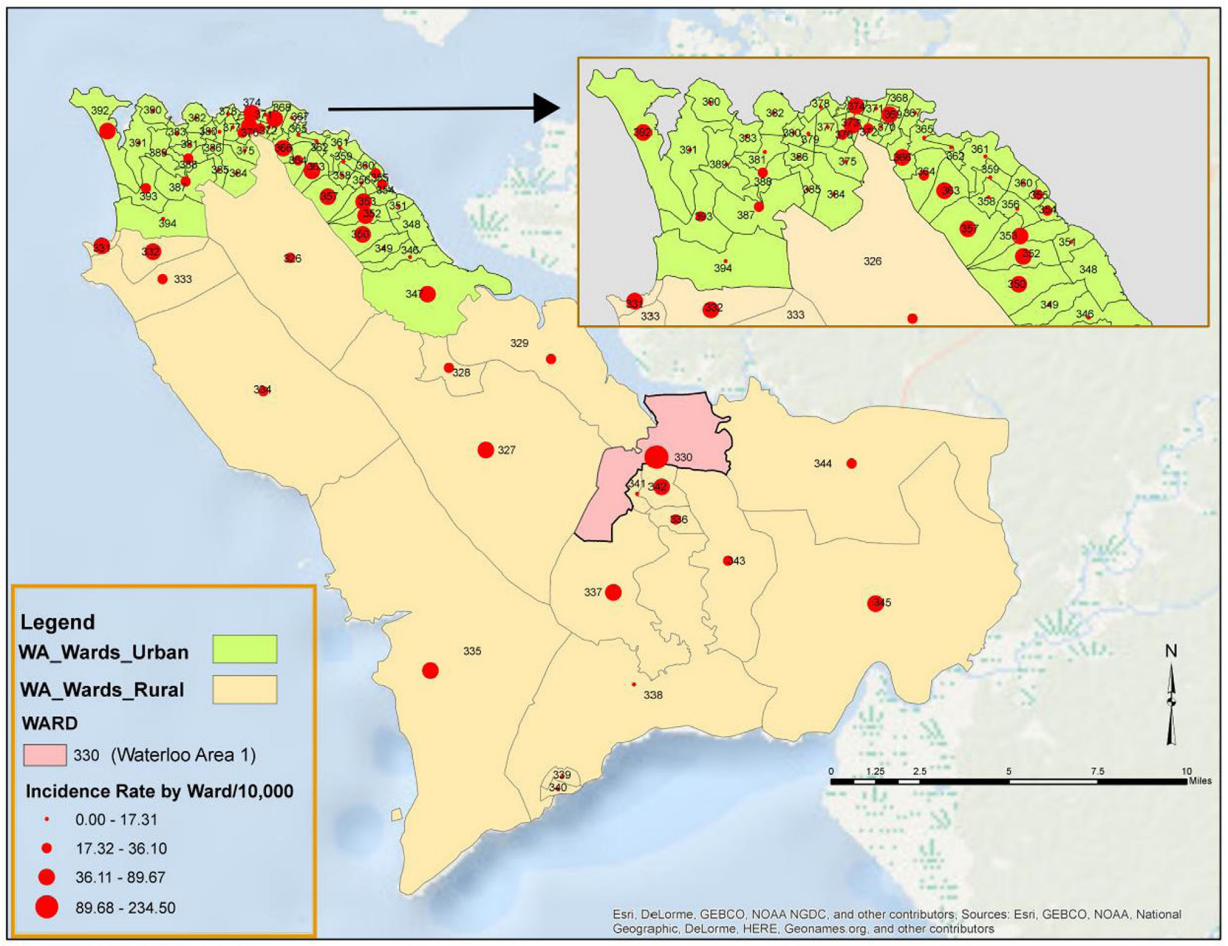
**Incidence rate per 1,000 population of Ebola virus disease by Ward in Western Area (WA): 2014–2015**.

All the 20 wards of WAR and 48 of the 49 wards in WAU recorded cases at various times during the epidemic. The only ward for which the viral hemorrhagic fever (VHF) database did not capture any documented EVD cases was Ward 351 (Bottom Oku village) in WAU. However, according to one of the DSOs in WA, cases were indeed reported from this ward even if not captured in the VHF database. The areas with the highest number of cases recorded in WAU were Allen Town I and II (Ward 347) with 213 cases and 78 deaths, Congo Water II (Ward 352) with 134 cases and 47 deaths, and Fourah Bay (Ward 369) with 103 cases and 20 deaths. The other wards in WAU reported cases ranging from 6 to 86 per ward. In WAR, the highest numbers of cases were reported from Waterloo Area I Area I (Ward 330) with 580 cases and 236 deaths, and the other 19 wards reported cases ranging from 7 to 83 per ward. Up to 634 cases (12.8%) and 201 deaths did not have their location or addresses specified. Figure 3 present the incident rate of EVD/1,000 population by wards in WA. Table 2 compares the proportion of confirmed cases that were death alerts (positive swabs) by district. Overall, the percentage of confirmed cases arising from death alerts was slightly higher in WAR (40.2%) than in WAU (31.7% WAU). The positive swabs represent cases confirmed after death, and not during illness.

**Table 2:** **Proportion of laboratory-confirmed cases by case status, districts, and the most affected wards in Western Area Rural (WAR) and Western Area Urban (WAU)**.

Variable	WAU/Freetown *N* (%)	WAR *N* (%)	Ward 330 WAR *N* (%)	Ward 347 (WAU) *N* (%)
Total lab-confirmed deaths	2,495	1,343	580	213
Confirmed swabs from dead alerts	791 (31.70)	540 (40.21)	236 (40.69)	78 (36.6)
Confirmed samples from live alerts	1,678 (67.25)	796 (59.27)	340 (58.62)	132 (62.0)
Missing status after disaggregation (swab/blood)	26 (1.04)	7 (0.52)	4 (0.69)	3 (1.4)

### Sociodemographic Characteristics of Cases

The ratio of male to female EVD cases, irrespective of the district of residence, was 1:1. About 22.4% of the cases were students, 14.6% were business communities, and 12.0% were children. An estimated 3.2% of the cases were health-care workers, and 0.4% were traditional healers. Most of the infected heath-care workers were from WAU.

### Illness Presentation and Exposure

Many of the cases presented with the typically known symptoms of EVD (Table 3). Very few cases reported unexplained bleeding. Only 20.1% of cases in WAU and 24.5% of cases in WAR reported being in contact with a known EVD suspect or patient prior to becoming ill. The 20% of cases with reported history of contact with a known EVD case in WAU indicated they had been exposed to a sick neighbor or a friend, while most of the cases with known history of contact in WAR had been in contact with close sick family or household members. Overall, qualitative information reviewed showed that transmission was mostly through contact with symptomatic individuals or dead bodies; however, information on the type of contact was missing and could not be quantified. Transmission to babies through breast milk was documented, and there were a few reports of potential sexual transmission.

**Table 3:** **Symptoms associated with EVD in Western Area (*N* = 2,720)**.

Symptoms reported^a^	Frequency of reporting	Percentage reporting
Fever	2,163	79.50
Fatigue	2,085	76.65
Anorexia	2,006	73.75
Headache	1,530	56.25
Muscle pain	1,497	55.04
Joint pain	1,456	53.53
Abdominal pain	1,379	50.70
Vomiting	1,352	49.71
Diarrhea	1,151	42.32
Difficult breathing	704	25.88
Conjunctivitis	633	23.27
Difficult swallowing	589	21.65
Unexplained bleeding	109	4.00
Bleeding from other sites (stool, injection sites, etc.)	2–6	0.07–0.22

*^a^Other minor reported symptoms included chest pain, jaundice, rashes, and hiccups*.

### Drivers of Transmission

The frequently reported drivers of transmission varied during the different phases, although some of them persisted through all phases of the outbreak. For example, the first half of the epidemic response was characterized by lack of or inadequate diagnostic and isolation capabilities, poor community health-seeking behavior, and management of ill health, including self-diagnosis, treatment of febrile illnesses using over-the-counter medication or herbal treatment, and a reliance on traditional healers. These factors were compounded by overcrowding as well as excessive population movements into and out of WA, and between WAU and WAR. Moreover, the initial phase of the outbreak response was characterized by a lack of resources, including inadequate capacities for surveillance that hampered early case detection.

The affected individuals and communities exhibited extreme fear: high-risk contacts and EVD-symptomatic individuals fled from one place to another. There were numerous reports of individuals exposed in WA taking refuge in their home districts to secure family support and care during their illnesses, while individuals exposed in other parts of the country with ongoing transmission fled from their districts to take refuge in WA, in an attempt to escape from the outbreak. Up to December of 2014, there were anecdotal reports of contacts and ill persons escaping quarantine homes from the other Provinces and coming to WAU/Freetown through Waterloo Area I Area I (Ward 330).

From January until the end of the outbreak, frequently observed and reported factors that exacerbated the rate of transmission included secret burials, high-risk contacts concealing exposure history or running away and getting lost to follow-up, communities hiding the sick and nursing patients from home, and a high number of positive cases derived from death alerts. Up until May 2015, there were reports of deaths or secret burials in quarantine homes despite daily monitoring of high-risk contacts for symptom developments. Some of the symptomatic contacts were reportedly taking antipyretics or herbal remedies to lower body temperatures before contact tracers checked them.

Some of the high-risk events that propagated the epidemic were attributed to a lack of community trust in the response and the fear of not having family members return back home after isolation. In WAU, many households with reported confirmed cases were found to be overcrowded, sometimes with up to 10 individuals in a room, many of whom were not related but sharing the establishment to share the cost of the rent. Between June and August 2015, there were a few reports of transmission to babies through breast milk and possible sexual transmission. There were widespread reports of families washing dead bodies even before calling the burial team to implement safe and dignified burial. Washing of dead bodies is a sociocultural practice engrained in these communities.

### Illness Outcomes

The overall case fatality for WA for the duration of the outbreak was 36.7%, although WAR registered an overall higher case fatality of 43.2%. All age groups were affected during the epidemic. When disaggregated by age, the data show that the highest fatality rate occurred among babies under the age of 1-year old (73.26%) and individuals over 65 years, recording about 69.76% dead (Table 4). Up to 31.7 and 42.1% of confirmed cases in WAU and WAR, respectively, were identified as death alerts.

**Table 4:** **Aggregated age-specific case fatality in WA, June 2014–November 2015**.

Age group (years)	Total cases (*N*)	% missing records		Dead (*N*)	Age-specific fatality (%)
		*N*	%		
<1	172	2	1.15	126	73.26
1–5	422	5	1.17	184	43.60
6–14	530	7	1.30	133	25.09
15–29	1,493	14	0.93	376	25.18
30–49	1,446	15	1.03	537	37.14
50–64	396	6	1.49	203	51.26
65+	291	0	0.00	203	69.76
Age not indicated	144	11	7.10	54	37.50
Total	4,954	60	1.21	1,816	36.66

## Discussion

Most previous outbreaks of Ebola were limited to rural areas (4) and were easier to contain. Thus, not many people anticipated that an Ebola outbreak could spread to an urban center and spin out of control to become a public health emergency of international concern. This study provides insights into the disease epidemiology in one of the worst hit urban settings during the first and unprecedented West African outbreak of Ebola in a low-income developing country. It also sheds light on some of the drivers and underlying factors for transmission that should be considered in similar context and comparable outbreak response efforts in the future.

Our study identified 4,955 probable and confirmed cases from the VHF database, a figure that is higher than what has been reported by the Sierra Leone MOHS, and the WHO by slightly more than 1,500 cases. The WHO reports of 3,449 cases (2). Even then, the data from the VHF database did not capture the earlier cases who were reported to be fleeing from Port-Loko into Waterloo Area I from around May 2014 when the outbreak probably spilled over into WA. This and the widespread reports of secret burials are possibly suggestive that even our figure is an underestimate. We believe that the discrepancies in the reported data and what could be the actual cases and death are attributed to gaps in the surveillance system as well existence of several disjointed databases, all of which were incomplete and could not be easily linked. Conceivably, the surveillance system was not able to detect all individuals with the clinical disease who intentionally evaded being tested or those who died and were buried without notification and/or laboratory confirmation. This is true for all phases of the response including the second and later phases of the outbreak response where reporting were based on laboratory-confirmed cases. Perhaps, these observations, also explain the higher numbers of probable cases reported in this study as compared to the official figures reported by MOHS and by WHO. These discrepancies in the reported surveillance data had earlier on been reported by Hui-Jun et al. (11) and the WHO ERT (1).

The disease epidemiology and insight into the underlying factors and drivers of sustained transmission highlight a number of lessons for the future. The outbreak in WA spilled over from ongoing transmission from the other Provinces. Despite the knowledge about ongoing EVD transmission in the other districts of Sierra Leone and the significantly increased population movements from all districts into WA for a variety of reasons, as observed by Chan (12), it appears that there was no anticipation that the outbreak could easily spill over into Freetown Municipality. As such, measures for preparedness and enhanced early warning and alerting system that should have been put in place to facilitate early detection was non-existent. It appears that the response was focused on containing the outbreak in the affected Provinces without measures to shield and protect the national Capital City and the surrounding suburb given the high population movements or at the minimum, conduct risk assessment and preparedness, including enhanced surveillance for early detection and swift response.

It is no doubt that the outbreak in WA was detected fairly late due to lack of diagnostic capabilities and the fact that symptomatic and high-risk contacts made every effort to conceal their symptoms. It was, therefore, impossible to determine the index case and the exact time when the outbreak was first introduced into WA districts. The intentional and persistent denial of the possible outbreak extension by community members, and failure to anticipate and appreciate possible implications by public health authorities and implement appropriate measures, resulted in the late detection of the outbreak in WA.

Unlike most previous EVD outbreaks in rural or semi-rural settings that were mostly limited in geographical scope, and tended to spread relatively more slowly (3, 13), the epidemic in WA was characterized by an unprecedented caseloads and deaths; with observed exponential increase in the number of cases especially in the initial phase of transmission that occurred between July and December 2014; widespread distribution of cases in all wards; and a prolonged duration of the outbreak. While the outbreak was widespread, intense transmission and higher death rates were observed in some of the areas like Waterloo Area I, Allen Town I and II, Congo Water II, and Fourah Bay. Some of these areas registered disproportionate higher numbers of positive swabs, in the range of 41–49%. Although the reasons for intense transmissions in these areas are not so clear, we attribute these higher caseloads to a number of factors that includes the presence of slums associated with overcrowding and not very organized settlements that provide safe havens for transmission and to hide from stigma and discrimination. The high number of positive swabs, ranging between 30 and 49% in some of the wards, further confirms the community’s behavior of concealing exposure history and disease symptoms and going into hiding only to be identified after death.

Our study, thus, corroborated the findings by Ansumana et al. (14), who reported that about 20% of individuals who died of EVD at the Ebola Treatment Center at Hastings Police Station in WA, died soon on arrival, meaning they presented for treatment late in the disease. Similar features characterized the outbreaks in the urban settings of Conakry, Guinea and Montserrado County of Liberia that experienced intense and prolonged transmission of EVD during this West African outbreak of Ebola (15, 16).

We did not examine the degree of spread from the capital city and urban setting of WA to other parts of the country in great details. However, we believe that the reported movements of the population, including of high-risk contacts and/or symptomatic individuals from WA into rural areas could have fueled transmission to other parts of the country. The diverse ethnicity of the population in the capital, which represents all ethnicities in the country, meant that the introduction of the disease in such urban setting was quickly followed by geographic widespread elsewhere, but also that ongoing transmissions in the districts could easily spill over into WA. Previous attempts to describe the transmission dynamics of Ebola in the urban cities of Guinea, Liberia, and Sierra Leone attributed the intense transmission and high caseloads to weak health systems and uncontrolled population movements (12, 17). Our study highlights the additional role of overcrowded settings coupled with fear, population behaviors, and lack of community trust in the response systems that manifested in the widespread efforts to conceal exposure history and disease symptoms, reports of secret burials, a high proportion of positive swabs, a high proportion of confirmed cases with no reported contact information, and persistent reports of high-risk contacts lost to follow up.

Lessons can be derived from the response to the extension of the same West African Ebola outbreak into Lagos, Nigeria, which is home to more than 21 million inhabitants. The response to this outbreak during the second half of 2014, clearly demonstrated some of the essential elements and precursor to effective outbreak response in any setting. Thus, with the right level of anticipation and awareness, proper preparedness, early detection, and swift response, an outbreak with high potential to explode because of the context can actually be limited and contained with minimal consequential morbidity and mortality (18, 19).

This study found that the proportion men and women affected by the disease, including deaths were about the same, probably reflecting the gender characteristics of the populations in the WA urban setting. In Pejuhun, a district in the northeastern part of Sierra Leone, however, females were more affected than males and transmission occurred mostly among family members (20). Similar findings were exhibited during the 2000–2001 Ebola outbreak (7). In contrast, transmission in WAU occurred mainly between neighbors and friends. This transmission pattern could be attributed to the fact that people were living in overcrowded shared accommodations to share and subsidize rental costs, and that the people living in the same rented accommodation in WAU were not necessarily blood related. The high numbers of individuals under 30 years of age, affected by the outbreak reflects the general demographic profile of the country which is very young (21).

This study did not examine differences in the epidemiology of EVD in an urban versus rural setting. However, as reported by Dietz et al. (22), a 10-fold increase in the weekly incidence of EVD in WAU/Freetown Municipality compared to the weekly incidence in other parts of the country was observed at the peak of the outbreak in Sierra Leone. This escalated transmission pattern is perhaps largely attributed to overcrowding and dense population, fleeing of high-risk contacts and sick persons from quarantine homes in the provinces, delayed health-seeking behavior, delayed detection of cases, secret burials, and the very high numbers of positive swabs. All these factors imply prolonged contacts and exposures to highly infectious symptomatic and or dead bodies, resulting in widespread transmission at community level, further aggravated by very poor infection control practices.

The above arguments are aligned to the findings by Ansumana et al. (17), who observed that ill persons and their family members routinely administered presumptive treatment of febrile illnesses and self-medicated with either over-the-counter medicine or herbal concoctions from traditional healers. The authors attributed the upsurge and exponential increase in the number of cases in Sierra Leone during the period of up to December 2014 to these behaviors. While these findings were also reported for WA, the ongoing EVD outbreak in surrounding Provinces was critical in sustaining and propagating local transmission in WA, particularly in Freetown and in Waterloo Area I Area I (Ward 330), the most densely populated cities as EVD high-risk contacts and patients fled quarantine homes from and into the provinces.

We report a relatively low-case fatality rate for the EVD outbreak in WA—a rate similar to those recorded in other areas of West Africa affected by this outbreak. Kucharski and Edmunds (23) have attributed the very low CFR for the West African Ebola outbreak to a failure to account for the delay between the onset of Ebola symptoms and disease outcomes (recovery or death). Our study provides additional insights derived from the outbreak context and community dynamics, and we attribute the very low case fatality for the outbreak in WA to a combination of factors. Thus, the diverse nature and ethnic representation of the population in WA, meant that some of the high-risk contacts and symptomatic individuals perhaps consciously returned to their home districts in the Provinces in search of nursing care and to be buried at home upon death; for some, fear and lack of trust in the response system may have resulted in high-risk contacts and symptomatic individuals fleeing into hiding; and possibly secret burials that were not captured in the data. If these were truly the case, then a significant proportion of the deaths attributable to EVD may have not been captured and accounted for, which may have resulted in the disease outcome variable skewed toward survival/recovery, depicted by the lower case fatality in WA.

In a similar vein, we believe that the very low proportion of confirmed cases with a reported history of contacts is partly due to the proactive concealment of exposure history by the affected communities and to inadequate surveillance system incapable of detecting cases early, especially in the early days of the epidemic. Hence, the delayed detection of cases and detection after death certainly played a critical role in the propagation and in sustained transmission. Delayed health-seeking behavior meant prolonged contact and exposure to others in the community and a community level amplification of transmission. This also confirms that the primary mode of EVD transmission in WA was person-to-person, as already reported by Park et al. (24) and Roels et al. (25).

We also note that this study registered a lower proportion of cases among health-care workers, a higher proportion of children infected, and a higher intra-community transmission, largely attributed to the behavior of community members. In contrast, a number of previous outbreaks in the rural settings, however, documented amplification of disease transmission in health-care settings (26–28).

### Study Limitations

Due to our study design and scope, which was limited to urban and peri-urban settings of WA, we could not compare and contrast EVD epidemiology in urban versus rural settings. The development and implementation of the surveillance system was slow and only became fully functional in the second half of the response. The negative behaviors of community members, such as concealing symptoms and conducting secret burials, could have resulted in a high number of missing individuals who died of the disease, especially early on in the outbreak. These people may not have been captured in the system, and neither do we have information about their characteristics. At the peak of the epidemic, the numbers of new cases and deaths overwhelmed the available capacities to systematically collect good data as per the surveillance protocol. Additionally, there was no capacity to adequately supervise the data collection. Consequently, the VHF database had missing variables, including exposure history which was largely derived from qualitative information. Additionally, it was not feasible to quantify the impact of the observed and reported drivers of transmission.

## Conclusion

This study confirms the challenges and difficulties of containing an infectious disease outbreak in an urban setting with dynamic and unresponsive communities, and in the context of limited or no public health resources. With an estimated 4,955 cases identified in WA over a 15-month period, WA experienced an unprecedented outbreak of Ebola in history, affecting equal numbers of men and female, and with the two, Freetown Municipality, and Waterloo Area I in WAU and WAR, respectively, becoming transmission hubs linked to cases elsewhere. While our study identified a lot more cases and deaths from the VHF database compared to what was reported by MOHS and by WHO, even the figures presented may be an underestimate of the actual numbers of cases and deaths attributed to EVD in WA during this epidemic period.

The outbreak in WA was detected late when it was already widespread. The delayed detection, in the absence of preparedness and swift response, resulted in the outbreak getting out of control. This unprecedented outbreak has been attributed to a number of factors; high population density, disorganized settlements, and slum areas, providing safe havens and hiding places for exposed and symptomatic individuals, uncontrolled population movements in and out of the WA, unfavorable behaviors among high-risk contacts and symptomatic individuals in communities such as hiding oneself and concealing disease symptoms, lack of trust in the response system, secret burials, and washing of dead bodies. The outbreak was characterized by intensive transmission at the community level. In WAU, most transmission occurred between non-related inhabitants of a shared accommodation and between neighbors. Additional potential routes of transmission, including through sex and breast milk have been reported.

The unique urban context and multi-ethnic nature of the WA, with linkages to all districts in Provinces elsewhere may have contributed to the sustained transmission and prolonged duration of the outbreak with infected and non-infected individuals moving in and out of the area. We were able to identify the significant role of fear, the lack of trust in the public health interventions, and the deeply ingrained sociocultural practices such as the washing of dead bodies—all of which led to propagation and sustained transmission, disease escalation, and widespread distribution of cases.

Anticipation of risks from an ongoing outbreak coupled with implementation of preparedness and mitigation measures in at-risk areas remains critical for early detection of disease expansion and swift response. This is even more critical if the at-risk areas are urban settings with potential for epidemic explosion following introduction. Response to future outbreaks in similar context should, therefore, be swift, comprehensive and build on the knowledge and understanding of the affected communities. Prioritization of strategic and targeted community mobilization and engagement activities to positively influence perceptions and practices is also important. Furthermore, good quality data are crucial for effective outbreak control and should be prioritized even more in the urban setting where community dynamics may complicate the disease epidemiology and control efforts. In such settings, steps should be taken to prioritize and ensure the immediate establishment of a robust active surveillance system able to capture all cases and generate relevant information for effective and timely intervention.

## Author Contributions

Conceptualized, designed, coordinated, and supervised the study: ML, JB, OO, ZY, TS, DK, NS, NW, AM, CD, and RA. Collected, analyzed, and reviewed output analysis: AS, JB, NW, K-BK, BM, MI, RC, MM, MK, JC, FD, FK-G, OO, CD, PF, and JB. Drafted the manuscript: ML, OO, JB, MI, TS, and K-BK. BK reviewed the analysis and contributed to the writing the paper. Read, provided inputs into the final draft of the manuscript, and agreed to be accountable for all aspects of the work: all authors. Gave final approval for publication of the manuscript: TS, BK, and RA.

## Conflict of Interest Statement

The authors declare that the research was conducted in the absence of any commercial or financial relationships that could be construed as a potential conflict of interest.

## Abbreviations

CDC, Centers for Disease Control and Prevention; CFR, case fatality rate; DSO, district surveillance officer; ETC, Ebola treatment center; EVD, Ebola virus disease; MOHS, Ministry of Health and Sanitation; MSF, Médecins Sand Frontières; NERC, National Ebola Resource Centre; OR, odds ratio; UNFPA, United Nations Population Fund; VHF, viral hemorrhagic fever; WA, Western Area; WAR, Western Area Rural District; WAU, Western Area Urban District; WHO, World Health Organization; ZEBOV, *Zaire ebolavirus*.
